# Birth Cohort Effect of the Mortality Rate from Congenital Heart Disease in Japan.

**DOI:** 10.2188/jea.13.274

**Published:** 2007-11-30

**Authors:** Morihiro Tajimi, Ritei Uehara, Makoto Watanabe, Izumi Oki, Toshiyuki Ojima, Yosikazu Nakumura

**Affiliations:** 1 Department of Public Health, Jichi Medical School.

**Keywords:** birth cohort effect, mortality, heart diseases, congenital, vital statistics

## Abstract

BACKGROUND: The mortality rate from congenital heart disease in Japan had decreased partly because the great improvement of medical treatment and care. Therefore, the patients who would have died of congenital heart disease in younger age in the past may be alive to be adult, and the number of adults with congenital heart disease might increase. If the management of such adults did not improve, the mortality rate from congenital heart disease might increase because of the increased number.

METHODS: Using the Japanese vital statistics from 1960 through 1999, we observed the time series of the mortality rate from congenital heart disease by age and sex. The birth cohort consisted of those who were bone in 5-year period starting in 1960. The mortality rate was the sum of the number of death in every five years divided by the population of the center year.

RESULTS: The transition of the mortality rate from congenital heart disease for age group 0 to 4 years was decreased since 1973. Other age groups showed decreased mortality rate since late 1960’s. The birth cohort analyses showed that the mortality rate of each birth cohort was decreased as time passed, especially the age group 5 to 9 years old and 10 to 14 years old.

CONCLUSION: Birth cohort effect of mortality from congenital heart disease exists in Japan.

Congenital heart disease (CHD) is one of the major fields of pediatric circulatory disease. The frequency is about 10 cases per 1,000 birth.^1^^,^^2^ Medical care for CHD has improved greatly, especially during the past two decades. Three main reason as follows were considered:^3^^-^^5^ First, non-invasive diagnostic procedures, such as angiography, ultrasonic cardiography using high frequency probe, and Doppler methods were introduced. Second, preoperative, and postoperative management was improved greatly. Especially, preoperative treatment was important because results of the surgical operation were much influenced by the preoperative condition. Third, various surgical methods were developed or modified, and the results of the surgical operation have been improved. Therefore, the patients who would have died of CHD in younger age in the past may be alive to be adults recently.

The ultimate goal of treatment, not only for CHD but also for all diseases, is for a patient to live out his/her natural life span. Improvement of treatment and medical care of CHD for young children might increase the number of adults with CHD. If there is no improvement of treatment and care for adult patients with CHD, the increased number of patients may introduce the elevation of mortality rate among these generations, and the ultimate goal is not reached. Because mortality rate from CHD of each age group at each year only shows the trend of the rate of each group, however, we observed the transition of the rate according to each cohort was grown up using birth cohort analyses. Thus, we evaluated the mortality rate from CHD using birth cohort analyses in Japan.

## METHODS

Using the Japanese vital statistics reports from 1960 through 1999, we observed the time series of the mortality rate of CHD by age-class and sex. International Statistical Classification of Diseases and Related Health Problems (ICD) have revived 3 times from 1960 through 1999; 1960 to1967 were seventh revision, 1968 to 1978 were eighth revision, 1979 to 1994 were ninth, and 1995 to 1999 were tenth revision. In this study, we used the International Detailed List Numbers 754 [congenital malformations of circulatory system] (IDC 7^th^ revision); 746 [congenital anomalies of heart] and 747 [other congenital anomalies of circulatory system] (IDC 8^th^ revision); 745 [bulbus cordis anomalies and anomalies of cardiac septal closure], 746 [other congenital anomalies of heart], and 747 [other congenital anomalies of circulatory system] (IDC 9^th^ revision); and Q20-28 [Congenital malformations of the circulatory system] (IDC 10^th^ revision) as CHD. The annual numbers of deaths from CHD during the observed 40 years were between 858 (1999) and 3,132 (1973).

**Figure 1.**
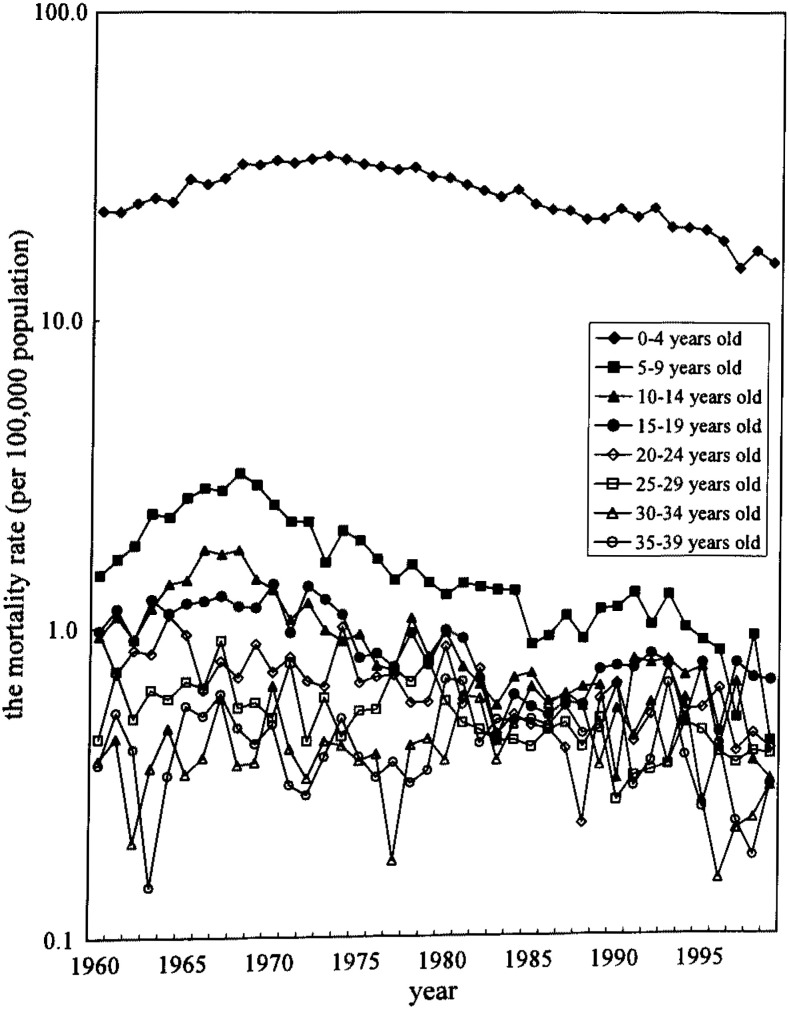
The mortality rate from congenital heart disease, 1960-1999, males, Japan.

**Figure 2.**
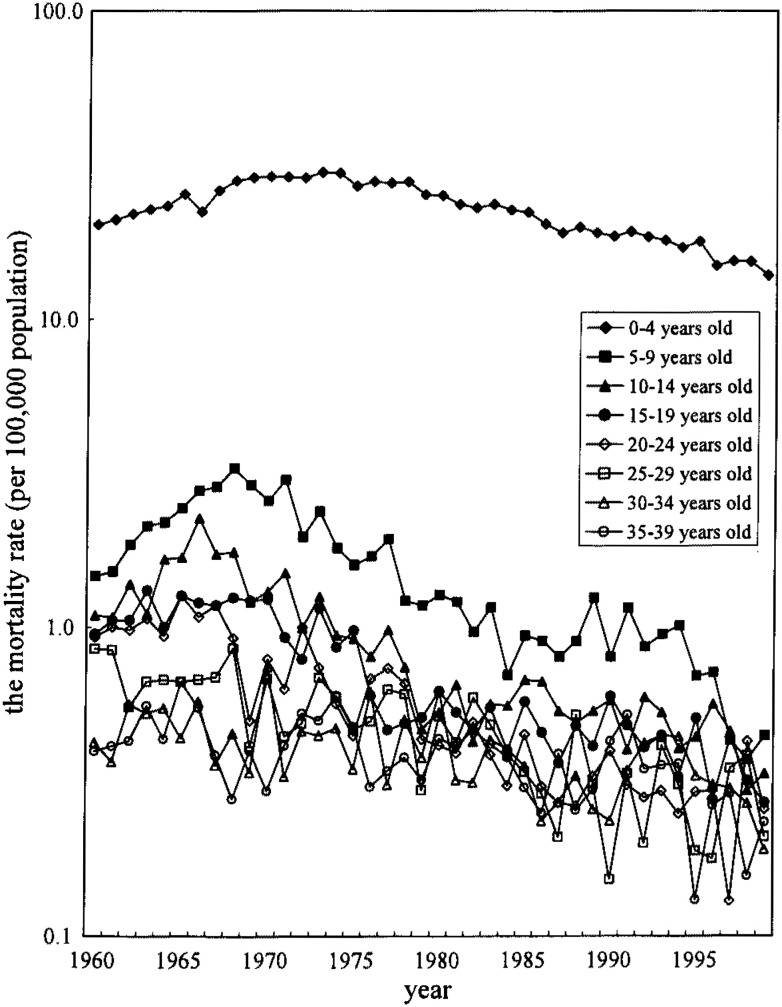
The mortality rate from congenital heart disease, 1960-1999, females, Japan.

The age categories were 5 years. The annual mortality rate was the number of death in every year divided by the population of the year for the observation of the yearly trend of the mortality as shown in Figures 1 and 2. The population of the 1970, 1975, 1980, 1985, 1990 and 1995 were obtained by a census, and others were by estimated population, which were used in the vital statistics shown as appendices of the vital statistics reports. The birth cohort consisted of those who were bone in 5-year period. The mortality rate per year in the birth cohort analyses was the sum of the number of death in every five years divided by the population of the center year multiplied by 5.

## RESULTS

As shown in Figures 1 and 2, the transition of the mortality rate from CHD of age group 0-4 years old was decreased since 1973 (male: 34.1 per 100,000 person, female: 30.0 per 100,000 person). In other age groups, although the mortality rates were much lower than those of the age group 0-4 years, the rate was decreased similarly to the age group 0-4 years after late 1960’s in both sexes except for 30-34 and 35-39 years old men which showed increasing mortality rate in 1980-94.

**Figure 3.**
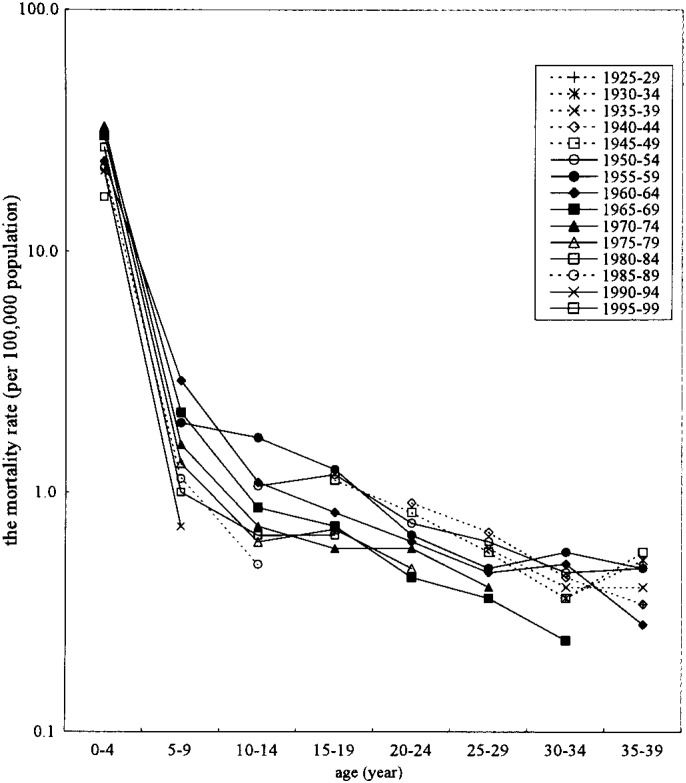
Birth cohort analyses of the mortality rate from congenital heart disease, 1960-1999, males, Japan.

**Figure 4.**
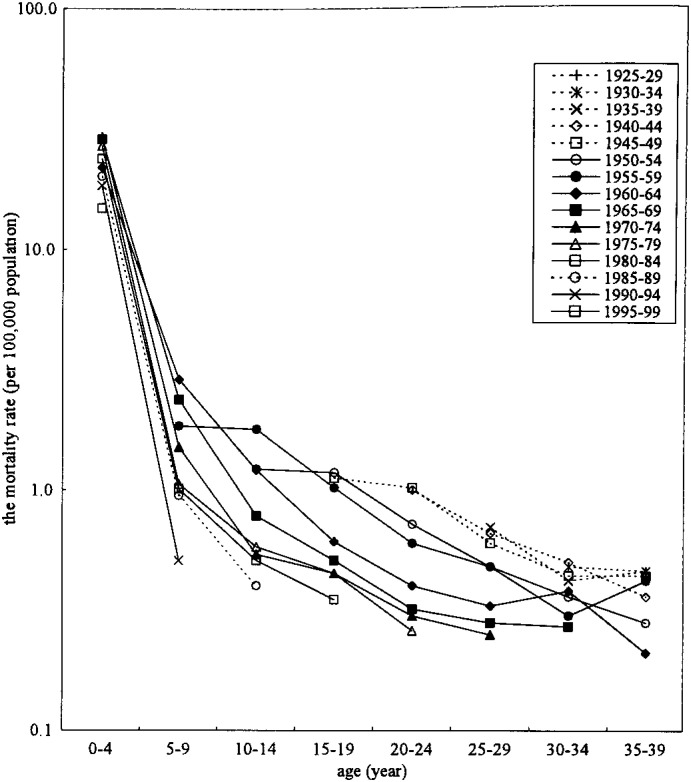
Birth cohort analyses of the mortality rate from congenital heart disease, 1960-1999, females, Japan.

The results of the birth cohort analyses are shown in Figures 3 and 4. In both sexes, the mortality rate from CHD was low among young generations in childhood. The mortality rate from CHD was also low among young generations in adulthood. However, although in both sexes, the mortality rate from CHD was also almost low among young generations in adulthood, the mortality rate slightly increased in 30-34 years old who were borne at 1955-1959 and 1960-64 in male, and 1960-64 in female.

## DISCUSSION

The mortality rates from CHD were declined continuously from late 1960’s, mainly because the medical care has been improved distinctly in many points mentioned above. This tendency was observed except for 30-34 and 35-39 years old men who showed increasing mortality rate in 1980-94. CHD in adulthood were only 1% in all cardiac mortality in adults in Japan. Takahashi^6^ described five reasons for CHD deaths in adulthood. First, they were not diagnosed because the symptoms were slight. Second, slight symptoms did not require treatments. Third, they were neglected by inappropriate medical care. Forth, their parents refused to treatment by economic and other reasons. Fifth, all the patients operated surgically did not have good post operative states. Thus they died of various complications such as arrhythmia and chronic circulatory disease. Recently, the birth rate of the baby with congenital disorder including CHD might be increased, because medical treatment or management was improved very much,^1^ and the medical care for CHD was improved greatly. Therefore, the mortality rate of CHD was decreased, and the decreased rate of childhood might bring the increased number of adult patients with CHD, no matter whether they have treated or not-treated, and the mortality rate of CHD might increase in adult age group. However, birth cohort analyses clearly showed the decline of the mortality rate of CHD according to the time passed. This phenomenon might implicate the true decrease of the mortality rate of CHD not only in childhood but also adulthood, and suggest the improvement of treatment and management of CHD for all age groups. The reason why 30-34 and 35-39 years old men showed increasing mortality rate in 1980-94 might be because the patients who were died in younger age in the past would live longer. Thus in further follow up is needed to confirm this speculation.

The limitation of this study is the validity of the causes of deaths. The vital statistics is based on the death certificates, and the causes of the certificates are questionable. Because the causes of deaths described on the death certificates are sometimes said to differ from the real diagnosis, our results might be different from the real features.

In conclusion, birth cohort effect of mortality from congenital heart disease exists in Japan.
